# Depression in the next generation is related with maternal behaviors: A cross-comparison by alternating rat’s mother care

**DOI:** 10.1371/journal.pone.0291952

**Published:** 2023-09-21

**Authors:** Ruixin Yong, Hongxia Chai, Lei Ran, Yuhao Li, Bei An

**Affiliations:** 1 The First Hospital of Lanzhou University, Lanzhou University, Lanzhou, Gansu, China; 2 School of Basic Medical Sciences, Lanzhou University, Lanzhou, Gansu, China; Texas A&M University College Station, UNITED STATES

## Abstract

This study investigated the potential impacts of depressive rats’ maternal behavior as an early life stress on the outcome of offspring as an adulthood. Offspring from the same mother were divided into two groups, half of them were fostered or remained by a depressive mother, and the other half remained or fostered by a control mother, respectively. The results showed that offspring fostered by depressive mothers presented significant depressive behaviors. Meanwhile, depressive mothers engaged in more grooming during the light cycle, but less off-the-pup behavior during the dark phase. In conclusion, offspring exposed to a postnatal depressive maternal environment developed a depressive-like behavior. Contrarily, postpartum maternal behaviors play an essential role, which might determine the outcome of the next generation. Furthermore, the appropriate timing of postpartum maternal caring sequences, which might eliminate prenatal stressful influences, was recognized and might be a promising approach for reducing children’s predisposition to mental disorders in their life time.

## Introduction

Five percent of the adult population in the world have experienced or are experiencing depressive related disorders [1]. Many researchers have reported that adverse experiences in childhood are associated with depression in adults [2–4]. This process is particularly susceptible during early critical periods, providing opportunities for environmental factors to impact the cognitive function of the brain [5]. However, the mechanisms of maternal environmental effects remain controversial including genetic factors, fetal programming, maternal style, insecure attachment, and parenting disorders [6–8]. Additionally, the Environmental Risk Longitudinal Twin Study has demonstrated that postpartum depression plays a mediating role in the intergenerational transmission phase [7]. Furthermore, parenting dysfunction particularly from the maternal part, plays a significant contribution for the development of depression and anxiety risk [9, 10], which disturbs the neuroendocrine stress reactivity by the HPA axis as peak timing of neurodevelopment in children [11, 12]. However, mothers are at great risk for perinatal depression [13–15], which might also lead to a range of adverse outcomes including premature delivery and developmental disorders [16, 17]. Collectively, depressive maternal care in rats is characterized by a disturbed sleep cycle, which implies that sleep-disturbed maternal rats during their gestational period may exhibit changes in maternal behavior [18]. These changes can impact cognitive and emotional functioning, leading to deficits in executive functions and emotional regulation in their offspring [19]. Similarly, mother-infant attachment levels, like maternal behavior as a moderator in the occurrence of depression of the next generation from early life stress impact, can be positive or negative [20, 21]. Moreover, a child born under a temporal disruptive environment and social dislocation resulting from caretaking has been reported and this has been a habitual factor manifested in couples [22].

Most studies have investigated the outcomes by directly intervening with pups [23] or solely studying maternal behaviors [24]. A difference in the maternal care patterns provided is a potential postnatal mechanism that may contribute to the final offspring performance. Additionally, maternal care in rats is a circadian rhythm [25]. Rodents’ grooming behavior is known to be highly sensitive to stress and anxiety [26]. Few studies have suggested that an increase in grooming by stressed mothers may be an indicator of elevated anxiety disorders in these rats [27]. So, maternal behavioral differences may be manifested through long-term daily observations with dams uninterrupted [27].

Collectively, the main objective of this study was to investigate how maternal care affects the development of offspring in rats that have been exposed to Chronic Unpredictable Mild Stress during gestation and lactation periods. Specifically, the study further provided a detailed analysis of the variability of postnatal maternal behavior in two groups—Intervention group (IM) and control group (CM) and examined how this behavior shapes the outcomes of their fostered offspring in four groups (IM-IM or IM-CM; or CM-IM or CM-CM).

It has been difficult to differentiate the impact of pregnancy on embryonic development from the influence of maternal behavior on offspring outcomes. In order to distinct the influences of maternal behavior during lactation from the effect of stress on embryonic development during pregnancy, we designed an “alternating mother care” strategy to control variables, which was only affected by the maternal behaviors compared with other control groups.

To determine the effects of maternal behavior influences on adult life of the next generation, the study conducted a series of depressive-like behavioral tests: a sucrose consumption test evaluated the anhedonia-like behavior; anxiety-like behavior were assessed by shorter locomotor inactivity time and low standing seconds on the open field test; serum cortisol level after acute stress presented the HPA axis ability; duration of inactivity behavior in the forced swim test exhibited the offspring’s struggle and despair. Notably, maternal behavior was monitored during the lactation period using a camera capture, and the sequences of these behaviors were found as certain behavioral sequences, which were analyzed. Depressive-like behavior in adult offspring were tested for assessing the outcomes when they were adulthood following postnatal day 80.

## Materials and methods

### Subjects and experimental protocol

Eight-week-old Sprague-Dawley rats (8 males and 16 non-mated females) were obtained from Lanzhou University, animal laboratory department, Gansu, China. These animals were bred in a house on a 12h:12 h light/dark cycle with free access to food and water under 20 ± 1°C and 55 ± 5% humidity. All males and females were fed separately in the experimental laboratory for one week for acclimatization purposes. All experimental procedures have been approved by the Lanzhou University Institutional Animal Care and Use Committee and permitted by the Guide for the Care and Use of Laboratory Animals (jcyxy20210604).

#### Experimental protocol of maternal groups

After the 7-day acclimatization period, two females and one male were put in one cage to mate for litters reproduction. Day 0 of the pregnancy (P0) was confirmed by locating sperms through a vaginal smear microscopy. All the rats in their gestational period were randomly assigned as control groups [Control Mothers (CM) n = 4] were reared by standard animal facility and intervention group (IM, Intervention Mothers, n = 4) were reared at Chronic Unpredictable Mild Stress (CUMS) environment. The intervention was sustained from P0 to postnatal day 21(PND21).

During pregnancy, maternal rats were fed in groups of four per cage until day 21 (P21). On P21, pregnant rats were moved to a separate cage for delivery. This cage was designated as the maternal-offspring cage and housed one maternal rat and eight pups until postnatal day 21 (PND21).

During the lactation period, the CUMS stimulation to maternal rats alone in the different cages for avoiding affecting their offspring. To control variables, CM were also taken out as the same frequency as IM group but without CUMS.

The purpose of this interventional protocol was to distinct the maternal behavioral effects on offspring adulthood bred by IM or CM.

#### Chronic Unpredictable Mild Stress in mothers

Maternal rats, which were removed from cages, in the intervention group (IM), were sustainably pressured by Chronic Unpredictable Mild Stress (CUMS) from P0 to PND21 until weaning. The time of intervention procedure began at 9:00 am and 9:00 pm every day for only half an hour lest separation anxiety. Mothers in the control group (CM) were stable and fed in a standard environment, and similarly removed but without any intervention.

To minimize the impact of stressors on development of the fetus in the uterine cavity, the study selected ultra-mild stressors for maternal rats. All 4 procedures, including (1) reversed light cycle, which involved covering the cage with shading at 9:00 am or using a lamp fixed 10 cm above the cage for illumination at 9:00 pm; (2) 45° cage tilt, which were feed the opposite side of nutrients to avoid feeding disturbances; (3) wet cage, which involved wetting the bedding to keep it completely damp using a watering can. The bedding was dried off before offspring returned to the cage during lactation; and (4) isolation, which involved removing maternal rats from their original cages for intervention while leaving the original cages unchanged, randomly conducted.

During the postnatal period, intervention procedures were performed in different laboratories to avoid potential effects on offspring caused by the intervention procedures.

#### Experimental protocols for offspring groups

**Fig 1:**
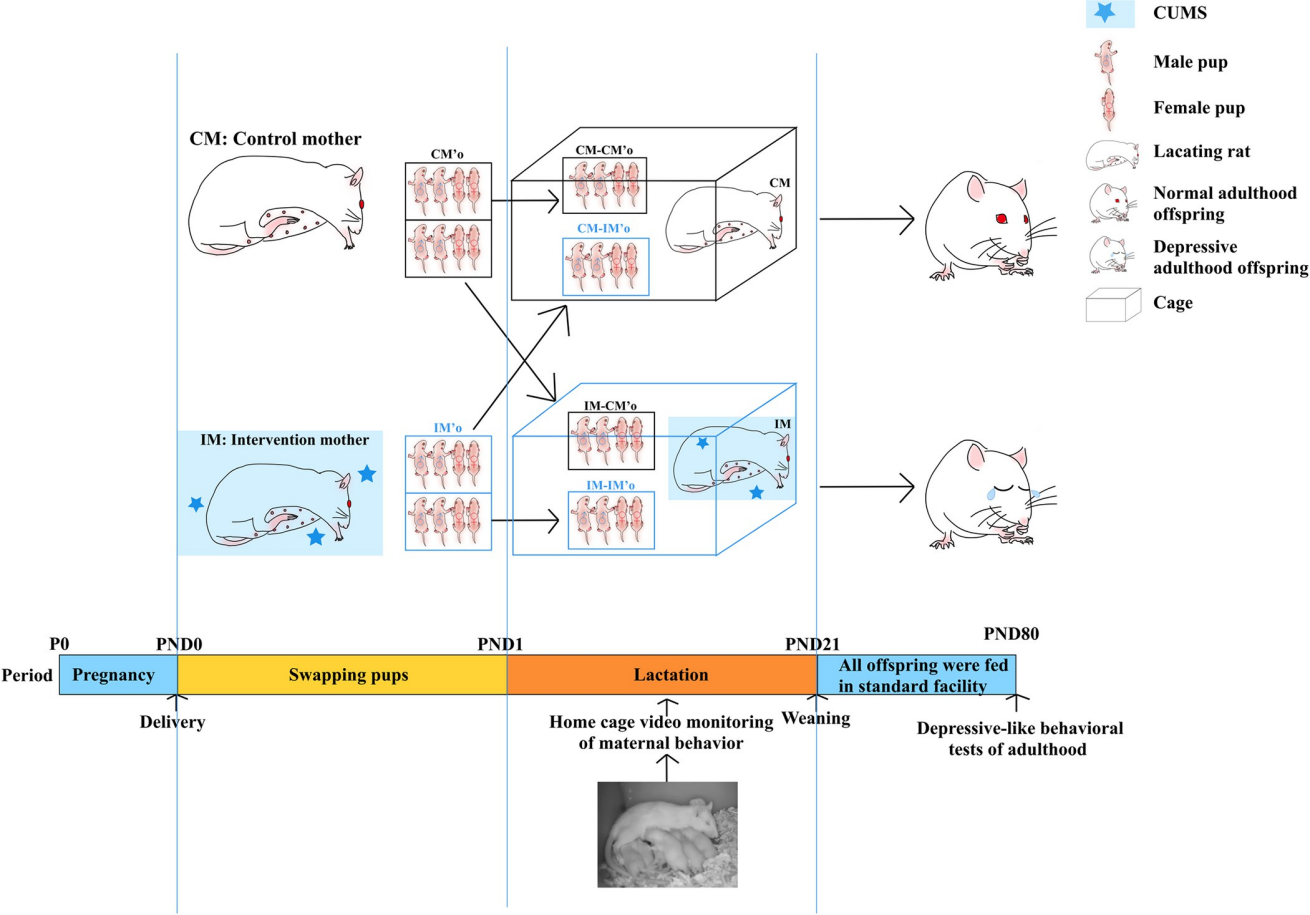
Procedure of the experiment with outcome. **Maternal** rats in their gestational periods in the intervention group (IM) were subjected to CUMS from day 0 (P0) pregnancy to postnatal day 21 (PND21), while maternal rats in their gestational periods in the control group (CM) remained intact. Half of the offspring remained with their biological mother and the other half were swapped and cross-reared between CM and IM groups. Offspring were divided into four groups: (1) IM-CM’o: the offspring were delivered by CM and reared by IM; (2) CM-CM’o: the offspring were delivered and reared by CM; (3) IM-IM’o: the offspring were delivered and reared by IM; (4) CM-IM’o: the offspring were delivered by IM and reared by CM. Maternal behaviors were monitored and recorded at PND11. All the offspring were weaned on PND21 and fed in standard facility until adulthood (PND80) to test the depressive-like behavior.

After the delivery process, pups from each cage were saved four males and four females on postnatal day 0 (PND0). IM cage was composed of four biological IM’s offspring (IM-IM’o), and four CM’s offspring (IM-CM’o). Pups, in the CM cage, composed of four biological CM’s offspring (CM-CM’o), and four adopted offspring delivered by IM (CM-IM’o). Meanwhile, the intervention and control group’s maternal rats that delivered within the same day were matched one by one. Offspring from the first cage were selected randomly and swapped with matching cage’s pups at a ratio of two males and two females (Fig 1).

After PND 21, all maternal rats were separated from their cages and their offspring were weaned. Then, all the offspring were fed eight per cage in a standard animal facility until adulthood (PND80) for depressive-like behavior tests. Maternal effects on offspring were tested by comparing differences of the depressive-like behavior tests among IM-IM’ o, CM-IM’o, CM-CM’o, and IM-CM’o.

### Depressive-like behavioral tests on offspring

To control the interferential effects of the tests, all the behavioral tests and cortisol level tests were conducted from 2:00 pm-4:00 pm during offspring adulthood (after or on PND80).

#### Sucrose preference test

A sucrose consumption test evaluated the anhedonia-like behavior by a protocol as previously described [28]. Offspring were supplied with two bottles filled with 2% sucrose solution or water. A two-bottle situation for 48h for acclimatization, foods and water were removed for 24h for deprivation purposes. To prevent any side preference, the location of the bottles was switched at one and half hours. Sucrose preference was calculated by a percentage of 2% sucrose solution intake of the total liquid intake (water + sucrose), according to the following formula:

$$\frac{surcose\;intake(g)}{surcose\;intake(g)+water\;intake(g)}\times 100\%$$

#### Open field test

Anxiety like-behavior were assessed by open field test [29]. A 60-W white bulb for illumination and a video camera (MI, Intelligent Camera 2K, China) for recording offspring behavior were fixed 1 m above the center of apparatus. The offspring were placed in the center of a black Plexiglass cage measuring 65cm x 65cm x 30 cm, and their behavioral activity was recorded using a video camera for 5 minutes. The duration of their inactivity and standing seconds were monitored and recorded. Standing was defined as the forelimbs being completely off the ground while using the hind limbs to stand, with the forelimbs resting at the side wall of the box. After each test, all boxes were wiped thoroughly with a 5% ethanol solution to remove the odor clues.

#### Forced swim test

The forced swim test was conducted as previously described [30]. Rats were immersed for 5 min in a Plexiglas cylinder (height 50 cm, width 25 cm), which was filled at a height of 40 cm with 30±1°C water. Behaviors were recorded with a video camera during the 5 min session, and the time spent floating, swimming and climbing was recorded. The definition of floating was making only the minimal movements necessary for the animal to keep its head above water and maintaining a vertical position of at least 10° from the surface. Inactivity behavior is defined as the offspring‘s head above water to float only making few movements necessary.

#### Serum cortisol

Serum cortisol level after acute stress shows the HPA axis ability of depression [11]. Serum cortisol was measured after the stimulation of the HPA axis by an acute stressor (forced swim). New blood samples were obtained from the tail vein within 3 min immediately. After 30 min of coagulation, serum was extracted by centrifuge at 3000 rpm for 5 min to obtain serum, which was stored at -80°C. Total cortisol was determined by an Enzyme Immunoassay kit (Rat Cortisol ELISA Kit, Catalogkit #MM-0574R1; MEIMIAN, China).

### Maternal behavior assessment

#### Home cage video monitoring

A method of continuous home cage video monitoring was described [23, 31]: each infrared camera was put above the cage for continuous home cage video record per cage. The frequency and duration time of any maternal behaviors per hour were analyzed.

#### Three maternal behaviors

Frequency of a certain behavior within one hour was defined as it occurred at this hour. If it continued on the next hour, it would be recorded as once as its starting hour. The accumulation of time with certain behavior in one hour, was the duration time above mentioned. Frequency and duration of three behaviors evaluated including grooming (maternal licking offspring’s feather and perineum to clean) (S1 File), breastfeeding (mothers were nursing in any position) (S2 File), and off-the-pups (any behavior without contact with pups) (S3 File). All three behaviors were completely independent.

All three behaviors occurred in a certain cyclic sequence were defined into normal cycling as ‘off-the-pups’ to ‘grooming’ to ‘breast-feeding’ cycle, and the reverse cycling as ‘breast-feeding’ to ‘grooming’ to ‘off-the-pups’ is. Alternate cycling was defined cycled between only two behaviors is, such as ‘off-the-pups’ to ‘grooming’ to ‘off-the-pups’ and so on. The count method of frequency and duration time on these cycles were recorded same as mentioned above.

### Statistical analysis

All data were presented mean ± standard error of the mean (SEM) per group and analyzed using spss26.0 (SPSS, Chicago, IL). Two-way analysis of variance (ANOVA) was applied to test for the behavioral tests of adult offspring. Dunn test was used for data that did not conform to the normal distribution. Three-way ANOVAs were applied for tasks employing multiple days of testing. Follow-up one-way ANOVAs were performed with significant post hoc Bonferroni comparisons reported. Three-way ANOVA was used to analyze the maternal video monitoring data. A significance threshold of α = 0.05 was used.

## Results

### Offspring behavioral tests

The main effect of maternal care on offspring was determined by comparing with IM-IM’o, CM-IM’o, CM-CM’o and IM-CM’o. Offspring with the same biological mother but reared by a different mother had different outcomes due to differences in maternal behavior sequences.

#### Sugar solution intake slightly higher consumed under CUMS maternal caring

**Fig 2:**
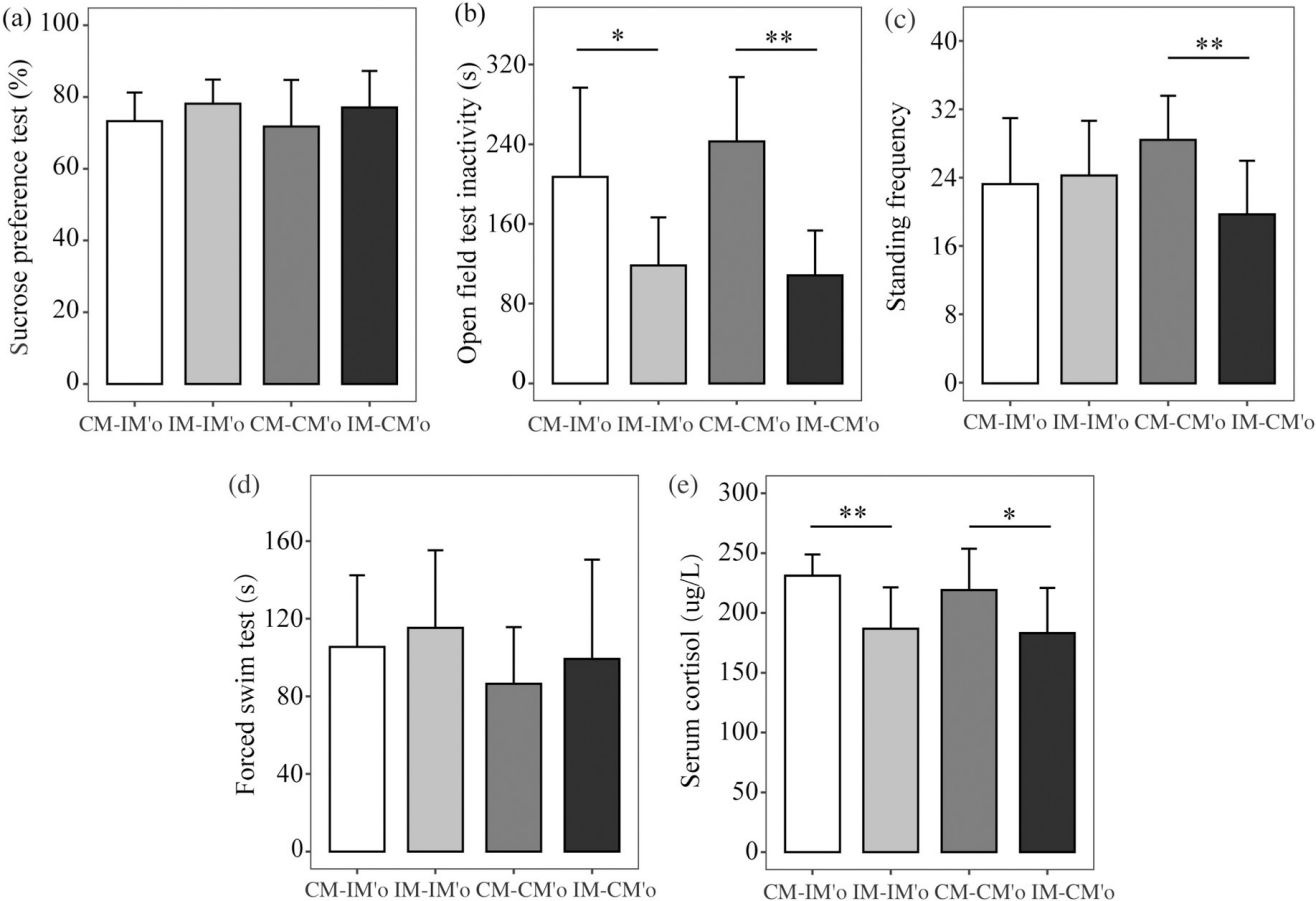
Depressive behavior of offspring tests. (a) sucrose preference test; (b) open field test inactivity; (c) standing frequency; (d) forced swim test; (e) serum cortisol. The four offspring groups were compared: (1) Intervention-Origin group (IM-IM’o); (2) Intervention-Foster group (IM-CM’o); (3) Control-Origin group (CM-CM’o); (4) Control-Foster group (CM-IM’o). Significant main effects of tests using a 2-tailed t-test, *P < 0.05, **P < 0.01.

The sucrose preference test assessed the anhedonia of offspring. In the offspring, sugar solutions intake of IM-IM’o were higher than CM-IM’o, which were sibling and reared by different maternal rats. Similarly, the consumption of IM-CM’o were higher than CM-CM’o.(F = 1.149, P = 0.339, Fig 2A).

#### Shorter duration of locomotor inactivity and lower standing frequency under CUMS maternal caring

The open field test was used to assess anxiety-like behaviors, with locomotor inactivity serving as the primary measure. Specifically, we recorded the duration of inactivity and frequency of standing within a 5-minutes period. In the offspring, which were raised by IM, both the IM-IM’o exposed to prenatal depression periods and IM-CM’o group free from prenatal depression periods, the duration of locomotor inactivity was shorter (F = 13.446, P = 0.000) (Fig 2B) and standing frequency was significantly lower (F = 4.754, P = 0.005, Fig 2C)than those in control group (CM-IM’o and CM-CM’o).

#### Longer duration of inactivity under CUMS maternal caring

During the forced swim test, we recorded the entire duration of inactivity behavior exhibited by the offspring to measure their struggle and despair. A 2-way ANOVA analysis of the forced swim test revealed offspring from IM-CM’o and IM-IM’o groups presented longer duration of inactivity than those from CM-CM’o and CM-IM’o (F = 1.266, P = 0.296, Fig 2D).

#### Lower level serum cortisol under CUMS maternal caring

Cortisol level after stress shows the HPA axis ability. After the forced swim test, we adopted the tail venous blood to test serum cortisol concentration immediately. Serum cortisol levels of offspring reared by IM (IM-IM’o and IM-CM’o) were significantly lower (F = 6.552, P = 0.001) than those cared by CM (CM-CM’o and CM-IM’o) (Fig 2E). There were no differences in offspring between IM-IM’o and IM-CM’o groups (P = 0.78) or CM-CM’o and CM-IM’o groups (P = 0.352), which were reared by same maternal rat.

### CUMS caused disrupted circadian rhythms in maternal behaviors

Depressive-like and anxious-like behaviors in adulthood offspring reared by IM group might be caused by care pattern.

#### Longer duration of breast-feeding during dark phase under CUMS

**Fig 3:**
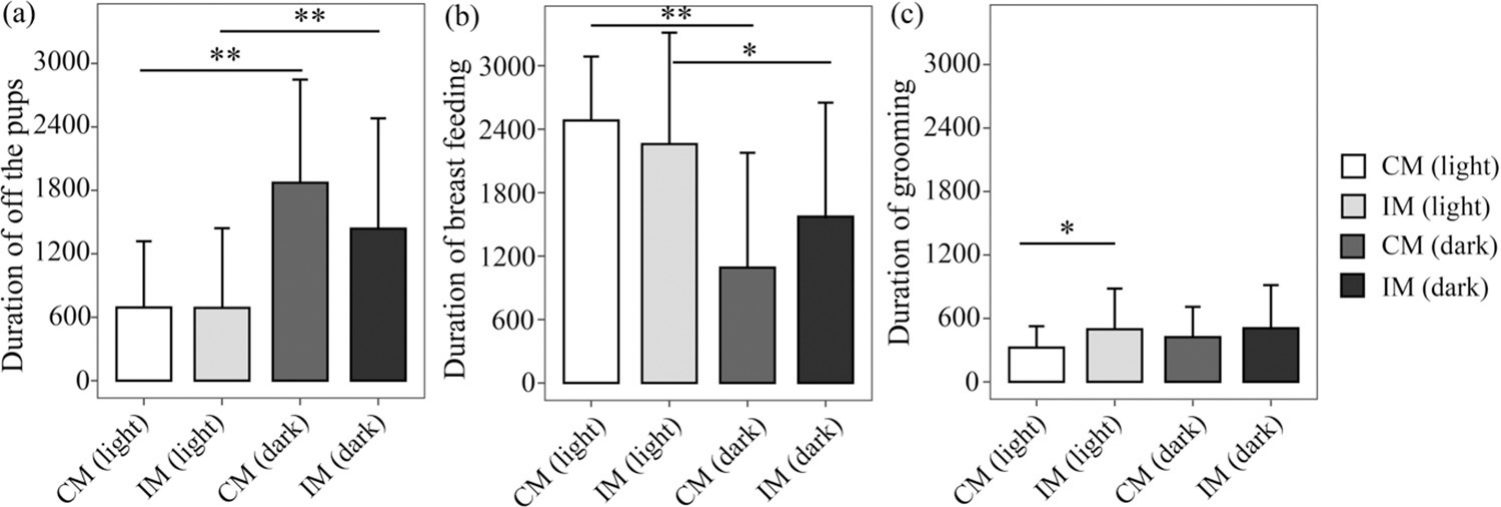
Durations of maternal behaviors which affected offspring during lactation was monitored by 24 h nest video. The mean time (seconds ± SEM) engaged in a maternal behavior for each hour over a 24 h period. Rats were housed in a 12:12 light: dark cycle, shaded areas indicate dark cycle and white areas indicate periods of light. Comparison of the maternal behavior in IM group and CM group: (a) Off-the-pups; (b) breast-feeding; (c) grooming.

Duration of all groups’ maternal rats off-the-pup behavior during dark phase are significantly longer than those during light phase (CM: Mean = 1728.00, SD = 1050.10; IM: Mean = 1437.24, SD = 1027.00 vs CM: Mean = 623.00, SD = 629.51; IM: Mean = 670.86, SD = 732.484; F = 13.203, P = 0.000; Fig 3A). Furthermore, the duration of breast-feeding during dark phase is significantly shorter than those during light phase (CM: Mean = 1107.94, SD = 1081.69; IM: Mean = 1486.20, SD = 1073.46 vs CM: Mean = 2272.50, SD = 863.50; IM: Mean = 2259.03, SD = 1053.46; F = 10.616, P = 0.000; Fig 3B).

**Fig 4:**
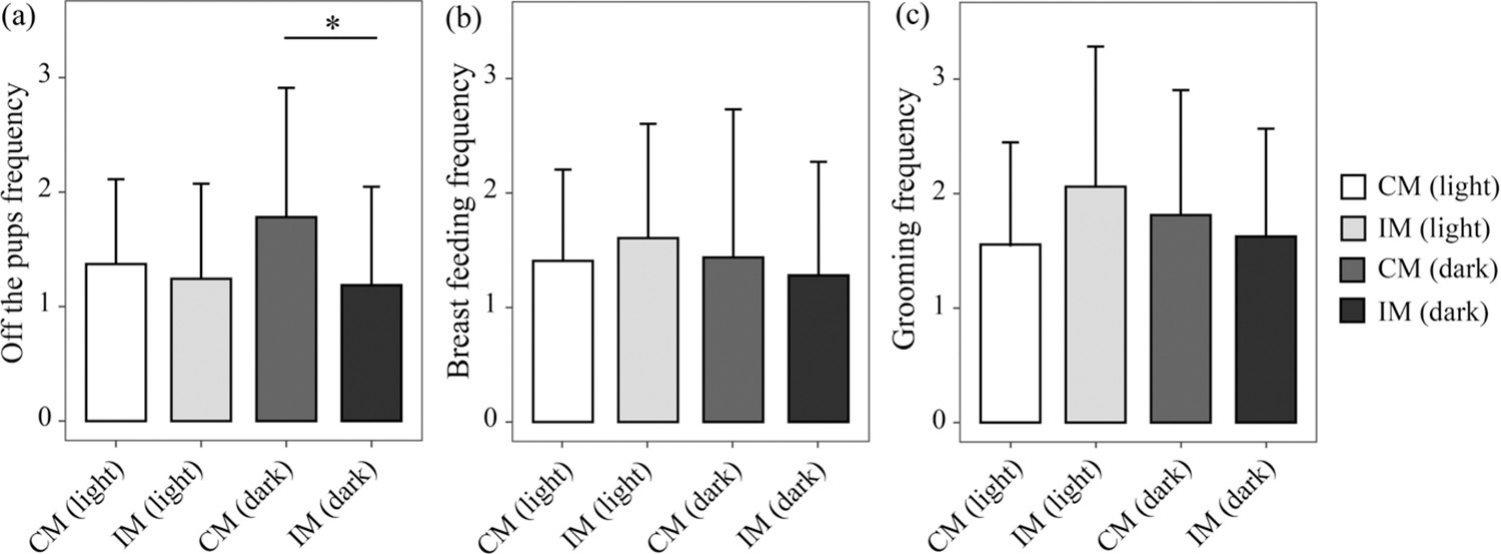
Frequencies of maternal behaviors which affect offspring during lactation was monitored by 24 h nest video. The frequency (number± SEM) engaged in a maternal behavior within one hour over a 24 h period. Rats were housed in a 12:12 light: dark phase, shaded areas indicate dark phase and white areas indicate periods of light. Comparisons of maternal behaviors in IM group and CM group: (a) Off-the-pups; (b) breast-feeding; (c) grooming.

Both the duration and frequency of off-the-pup in IM group is lower than those in CM group during dark phase (Figs 3A and 4A). Duration of the breast-feeding of IM in dark phase is longer than those CM groups. (CM: Mean = 1107.94, SD = 1081.69; IM: Mean = 1486.20, SD = 1073.46 vs CM: Mean = 2272.50, SD = 863.50; IM: Mean = 2259.03, SD = 1053.46) (Fig 3B). The frequency of breast-feeding in IM group is higher than that in CM group during light phase and opposite case during dark phase (Fig 4B).

#### CUMS increases duration and frequency of grooming behavior during light phase

Duration of grooming behaviors in IM group is longer than that in CM during light cycle. Higher frequency of grooming behavior was observed in IM group than those in CM group during light cycle (Figs 3C and 4C).

#### CUMS caused more ‘reverse cycling’ sequence occurred in IM group

**Fig 5:**
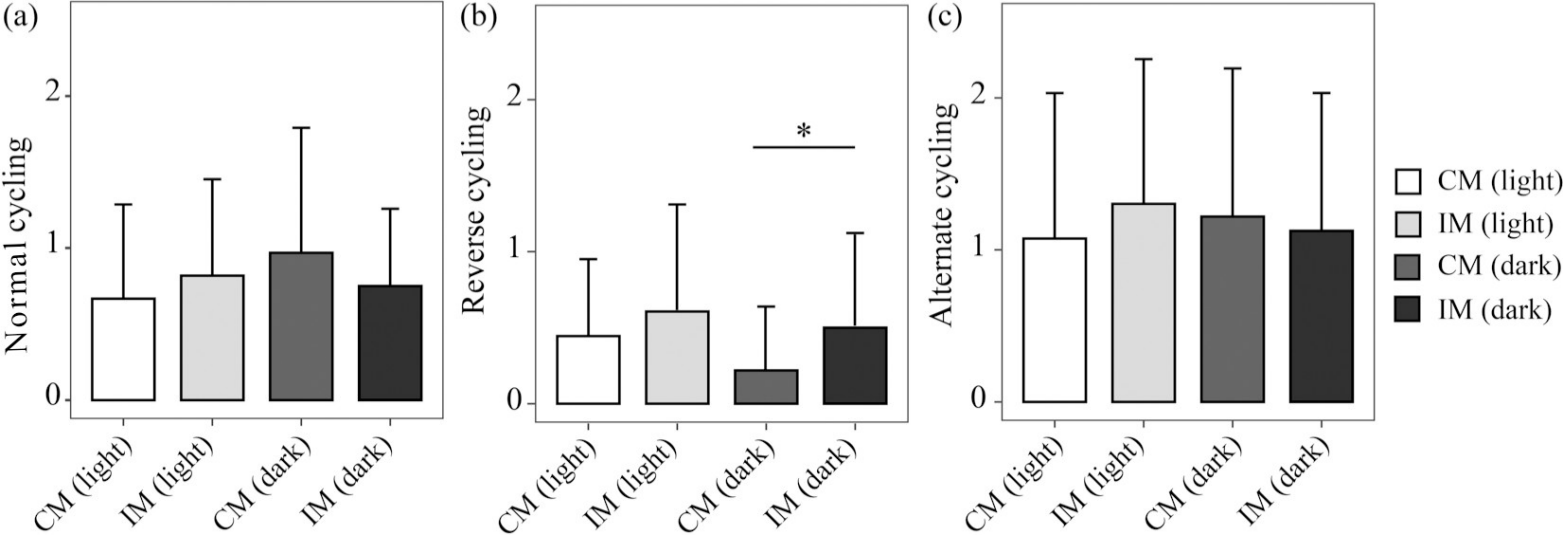
Cycling of maternal behaviors that affected offspring mental health during lactation were monitored at 24 hours nest video.

There was no significant difference about behavioral sequences between the light and dark phases. Alternate cycling was dominate (CM group: Mean = 24.00, SD = 8.00 vs IM group: Mean = 28, SD = 4.36), secondary was normal cycling (CM group: Mean = 17.33, SD = 3.06 vs IM group: Mean = 17.33, SD = 2.31) (Fig 5). Frequency of reverse cycling occurred in IM group was obviously higher than those in CM group during dark phase (CM group: Mean12.33, SD = 5.86 VS IM group: Mean = 6.33, SD = 1.15; Fig 5B).

All of the three behaviors including off-the-pups, grooming, and breast feeding occur as a certain order. (a) Normal cycling was defined as off-the-pups-grooming-breast feeding cycle. (b) Reverse cycling was off-the-pups-breast-feeding-grooming. (c) Alternate cycling was between any two behaviors. The frequency (number± SEM) engaged in these three behavioral sequences for each hour over a 24 h period. Rats were housed in a 12:12 light: dark cycle, shaded areas indicated the dark phase and white areas indicated periods of illumination. Comparison the maternal behavior in the IM group and CM groups.

## Discussion

In this study it was found out that all the adulthood offspring presented adverse outcomes, which reared by IM exposed to CUMS maternal behaviors, no matter who experienced gestational exposure to CUMS (IM-IM’o group) or not (IM-CM’o). Notably, all the adulthood offspring reared by CM presented healthy behaviors (both CM-IM’o and CM-CM’o). This might indicate that healthy maternal care could serve as a therapeutic method to improve the offspring mental health.

### Depressive outcomes on the offspring reared by CUMS maternal rat

Collectively, depressive outcomes were confirmed in this research that offspring, both IM-IM’o and IM-CM’o, which were born from different mother and reared by same CUMS mother, presented similar behaviors featured more depression (Fig 2). It might indicate maternal behaviors have a mediating effect on offspring mental development [21].

In this study, the offspring experienced lactational environment exposed to CUMS, cortisol levels were lower after acute stress which indicated that the HPA axis development was disturbed since prenatal and postnatal periods were vulnerable and vital windows for HPA axis neurodevelopment [5, 11, 12, 32]. Moreover, offspring cared for by the IM (IM-IM’o and IM-CM’o) exhibited shorter durations of locomotor inactivity in the open field test, and a trend towards longer durations of inactivity in the forced swim test, compared to offspring reared by the CM (CM-CM’o and CM-IM’o). These results indicated depressive-like manifestations in adulthood offspring reared by IM.

### Maternal behavioral significant roles in the next generation

Maternal grooming in rodents can be a stimulation of somatosensory which supports neurodevelopmental outcomes [33] and regulates the central nervous system dysfunction from separation effects [34–36]. High frequent maternal grooming helps children build strong connectivity with the right dorsomedial prefrontal cortex [33]. Furthermore, grooming is entirely maternal spontaneous behavior in rodents for cleaning pups up and helping in the urination process and regulating temperature as a somatosensory input [35].

**Fig 6:**
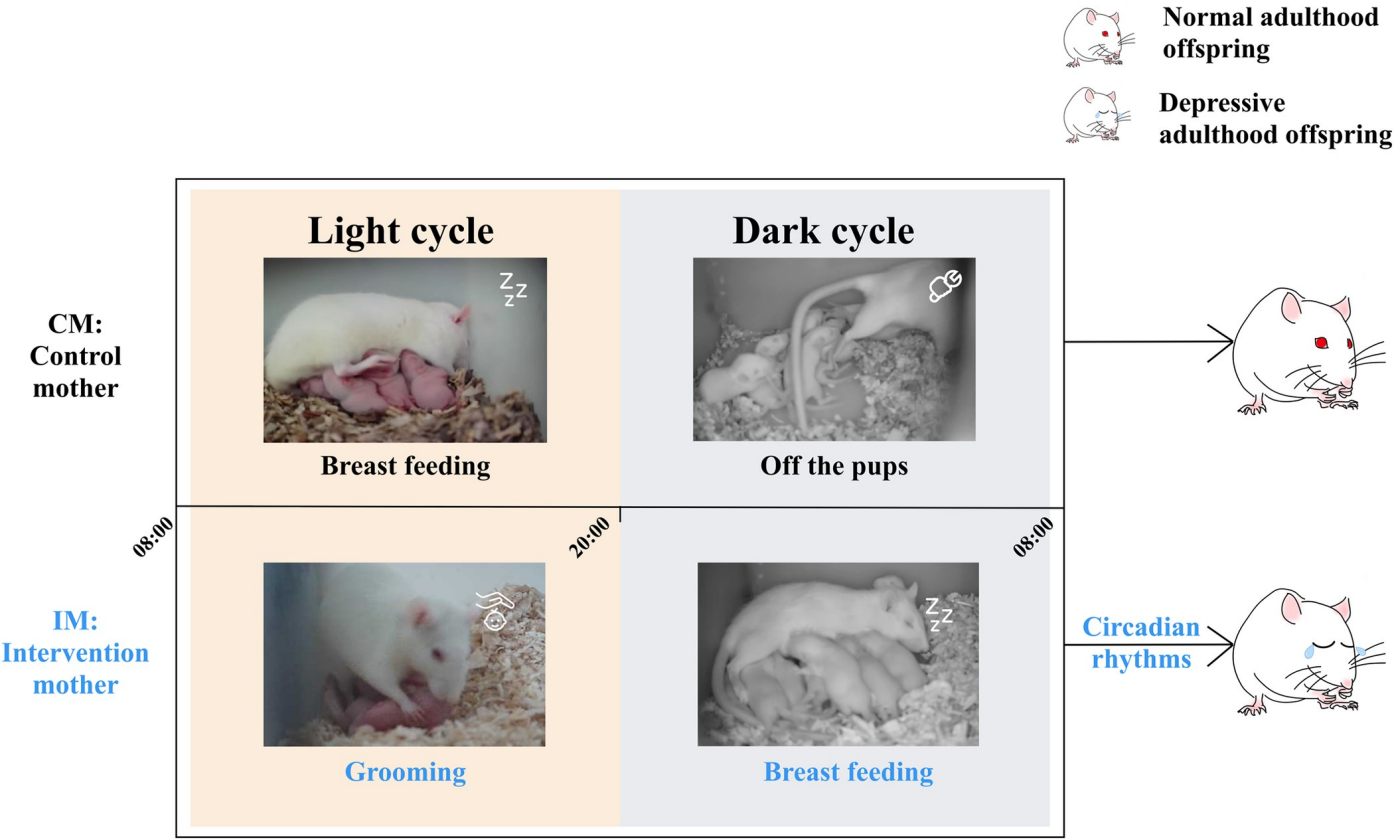
Concept graphs of circadian rhythms of control mothers and stressed mothers. Images of rats were collected on the home cage infrared monitoring device which were different maternal behaviors described in the Result section. Mothers in the CM tend to more breast-feeding and less grooming in light cycle compared with IM. Mothers in the IM showed a tend who are less off-the-pups and more breast-feeding in dark cycle. According to the definition and observation of these behaviors, breast-feeding was considered to be a manifestation of inactivity, and off-the-pups and grooming was activity.

However, the increase in grooming by stressed mothers also may be indicative of elevated anxiety in these rodents for lactation mood disorders [27]. Moreover, mothers in the IM group tend to present decreased activity during the dark phase and increased activity during the light phase, which proved the stressed maternal rats had disrupted circadian rhythms (Fig 6). Badly, excessive grooming behaviors during the light phase by CUMS maternal rats in the IM group could disturb their offspring’s rest due to their nocturnal habits and disrupt their natural rhythms, potentially resulting in a predisposition towards mental disorders in the offspring life time.

Based on certain main effects of the circadian cycle, CUMS maternal behaviors presented an inactive trend, during dark phase: it was characterized with shorter duration of off-the-pup and less frequency (Figs 3A and 4A); and longer duration of breast-feeding and less frequency. And, there is more activities during light phase (Figs 3B and 4B). Hence, the offspring reared by IM may be impaired their cognitive and emotional development [37]. And timing of maternal care is very essential not frequency. Of note, the infant hippocampal corticoid receptor system of rat is altered by maternal separation with enhanced HPA responsiveness to stress and a stress response prolonged [34, 36]. Furthermore, high frequent off-the-pup behaviors, leaving pups lack of warm, breast and attachment and causing separation anxiety, in IM group are also a risk factor leading to depression in offspring developmental stages including [38–40].

### Certain sequences of maternal behaviors——a new vision

Some research and investigative work have reported that certain sequences of maternal behavior can disturb offspring as an early stress [41, 42]. In our study, three certain sequences were defined: ‘normal cycling’, ‘reverse cycling’ and ‘alternate cycling’. The normal cycling occurs as from ‘Off-the-pups’ to ‘grooming’ to ‘breast-feeding’. Grooming after separation can greatly calm anxiety and timely clean up the excreta of offspring, which we could see as main sequence is normal cycling in normal groups.

**Fig 7:**
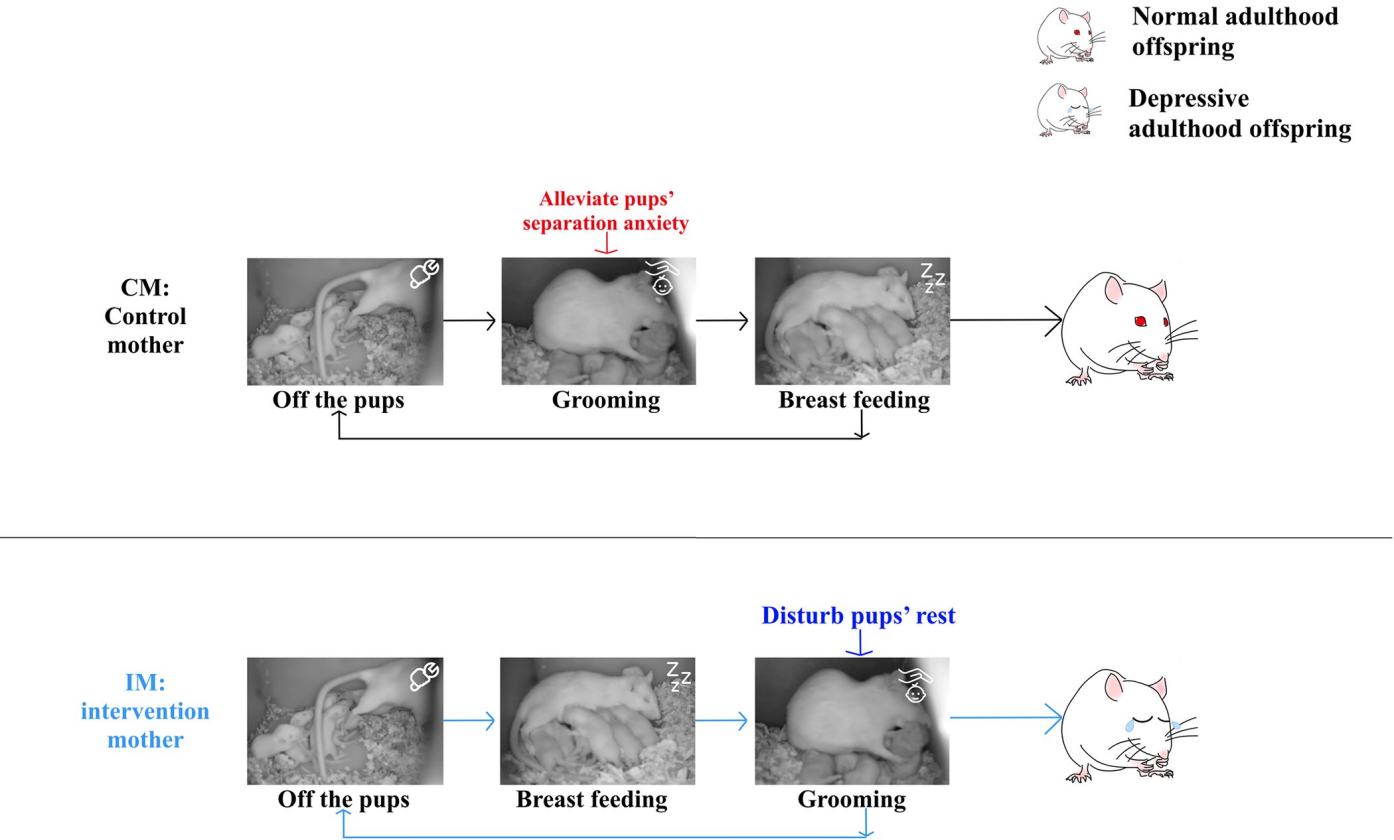
Concept graphs of cycling sequence of maternal behaviors of control mothers and stressed mothers. Images of rats were collected on the home cage infrared monitoring device which were different maternal behaviors described in the Result section. Maternal behavior cycles in a certain order. Normal cycling was sequent as off-the-pups to grooming to breast feeding. Reverse cycling was off-the-pups to breast-feeding to grooming. Normal cycling was main pattern to the normal mothers, but there was more reverse cycling in the IM. In the normal cycling grooming after separation can greatly alleviate anxiety. And from reverse sequence, grooming after breast-feeding disturb the rest on offspring in IM.

On the contrary, more ‘reverse cycling’ occurred in IM group compared with CM group during the dark phase (Fig 5). From this sequence, the offspring cannot get enough rest, disturbing the offspring, when grooming after breast-feeding. (Fig 7)

Notably, depressive maternal cares in rats are characterized disturbed sleep [19], sleep-restricted pregnant rats might have change in maternal behavior [18]. Reverse cycling occurs as from off-the-pups to breast-feeding to grooming. In contrast to some previous conclusions that higher frequent or longer duration of baby care with mental health [43], we do find that maternal behavior from IM group delineated by higher frequent and shorter duration breast-feeding during the light phase but lower frequent activity and longer duration breast-feeding behavior with adverse outcomes.

Hence, it is not only the frequency or duration of breast-feeding but their natural rhythms affect offspring mental health. This might explain bias in previous research due to timing neglected [43–45]. It can be explained that higher frequent and longer duration of grooming behaviors and more trends of off-the-pups at light phase in IM group could disrupt neurodevelopmental outcomes and lead to depressive-like behavior of offspring, in consistent with that worse maternal care ruined entrainment of the central clock parameters in the rat during the early developmental stages [25], because their timing is disruptive to the circadian rhythms [46].

### Envision

In addition to the maternal behavioral effects indicated by this paper, some of other results deserve attention. Perinatal depression has various effects on the mental health of the offspring, but it is difficult to distinguish between the influence of embryonic development in antepartum and maternal behaviors in postpartum. Even, these effects always are co-occurrence. In order to eliminate the effect on embryonic development, some offspring were reared by maternal rat from CUMS group. And maternal behavior in IM groups featured disrupted rhythms compared with CM group, which supports that parenting behaviors play detrimental roles in the development of depressive symptoms [32].

At the same time in our case, adulthood outcomes and features in offspring CM-IM’o, who suffered from prenatal stress in IM group, did not present depressive behavior. There was no significant difference from the CM-CM’o group and significant difference from IM-IM’o. CM-IM’o were born from intervention cage, but reared by healthy mother. The affection by depressive prenatal environment might be mitigated by CM’s proper maternal behaviors as their foster mother [25]. Many reports attributed this positive effect to postpartum care or child-care [46, 47].

In terms of prenatal effects, the mechanism is very complex and unclear. Some schools believe that the mother’s HPA axis involves in the cortisol level which is a strong correlation between mother and fetus [48]. However, existing theories are not sufficient to explain how proper maternal behaviors eliminate embryonic effects. This may be related to neuroplasticity in the development of children [49]. Based on this study, we have begun to further investigate the embryonic effects of CM-IM’o, such as monitoring the cortisol level in umbilical cord blood of IM or detecting the fetal brain cells. Similarly, we also look forward to the in-depth discussion of this issue by yours.

## Conclusion

In conclusion, in our study we have found out that maternal rats in the IM groups, who were subjected to continuous CUMS intervention, exhibited different caring behavior patterns during the lactation period compared to those in the CM group caring behavior patterns. The ‘reverse cycling’ sequence of maternal behaviors observed in the IM group may have a significant effect on offspring as an early life stressor, potentially leading to dysfunction of the HPA axis in offspring with depression-like behaviors. The study suggests that the better maternal behavioral pattern may benefit the children mental healthy development. However, further longitudinal research is needed to validate this claim and evidence.

## Supporting information

S1 File Grooming. This is a maternal behavior video captured by infrared camera in continuous home cage video monitoring: maternal rat is licking offspring’s feather and perineum to clean.
(MP4)

S2 File Breastfeeding. This is a maternal behavior video captured by infrared camera in continuous home cage video monitoring: maternal rat is nursing in any position.
(MP4)

S3 File Off-the-pups. This is a maternal behavior video captured by infrared camera in continuous home cage video monitoring: maternal rat is doing any behavior without contacting with pups.
(MP4)

S1 Data (XLSX)
